# Naofucong Ameliorates High Glucose Induced Hippocampal Neuron Injury Through Suppressing P2X7/NLRP1/Caspase-1 Pathway

**DOI:** 10.3389/fphar.2021.647116

**Published:** 2021-05-20

**Authors:** Guangchan Jing, Huanyuan Wang, Fengwei Nan, Yuqin Liu, Mengren Zhang

**Affiliations:** ^1^ Department of Traditional Chinese Medicine, Peking Union Medical College Hospital, Peking Union Medical College and Chinese Academy of Medical Sciences, Beijing, China; ^2^ Acupuncture and Tuina Department, Qilu Hospital of Shandong University, Jinan, China; ^3^ Department of Endocrinology, Kaifeng Hospital of Traditional Chinese Medicine, Kaifeng, China; ^4^ Department of Cell Resource Center, Institute of Basic Medical Science, Peking Union Medical College and Chinese Academy of Medical Sciences, Beijing, China

**Keywords:** naofucong, pyroptosis, high glucose, hippocampal neurons, TCM

## Abstract

P2X7/NLRP1/caspase-1 mediated neuronal injury plays an important role in diabetic cognitive impairment and eventually inflammatory cascade reaction. Chinese herbal compound Naofucong has been mainly used to treat cognitive disorders in Traditional Chinese Medicine The present study aimed to investigate whether its neuroprotective effects might be related to the inhibition of P2X7R/NLRP1/caspase-1 mediated neuronal injury or not. In this study, high glucose-induced HT22 hippocampal neurons were used to determine Naofucong-containing serum neuronal protective effects. Lentiviruses knock out of TXNIP and P2X7R was used to determine that protective effects of Naofucong was related to inflammatory response and P2X7/NLRP1/caspase-1 mediated neuronal injury. NAC was also used to inhibit oxidative stress, so as to determine that oxidative stress is an important starting factor for neuronal injury of HT22 cells cultured with high glucose. Naofucong decreased apoptosis, IL-1β and IL-18 levels in high glucose-induced HT22 hippocampal neuron cells. Naofucong suppressed NLRP1/caspase-1 mediated neuronal injury, and P2X7 was involved in process. HT22 cells cultured in high glucose had an internal environment with elevated oxidative stress, which could promote neuronal injury. The current study demonstrated that Naofucong could significantly improve high glucose-induced HT22 hippocampal neuron injury, which might be related to suppress P2X7R/NLRP1/caspase-1 pathway, which provides novel evidence to support the future clinical use of Naofucong.

## Introduction

Diabetes mellitus (DM) is a kind of glucose metabolic disorder involving multiple system damage (Xu et al., 2013). And, DM can cause cognitive decline, manifested as impaired attention, motor speed, executive function and verbal memory, which seriously affects people's health (Palta et al., 2014). Thus, to explore pathogenesis and corresponding therapeutic targets of diabetic cognitive impairment is an important measure to treat diabetic complications. Many studies have shown that diabetes is an autoimmune and low-grade inflammatory disease, and that inflammatory responses play an important role in diabetic cognitive dysfunction (Romeo et al., 2012). There are many factors and pathways in the damaged neurons of diabetic patients that can cause the inflammatory cascade, and P2X7/NLRP1/caspase-1 is one of the most important pathways.

P2X7/NLRP1/caspase-1 pathway is an important pro-inflammatory pathway, which depends on caspase-1 activation and followed by inflammatory cascade. Under endogenous and exogenous stimulation, apoptosis-related spot-like protein (ASC) activates pro-caspase-1 (Doitsh et al., 2014). It interacts with nucleotide binding oligomerization domain-like receptor protein 1 (NLRP1), which is involved in formation of inflammosomes and activation of caspase-1 (Tan et al., 2015; White et al., 2017; Park et al., 2018). Activated caspase-1 induces the activation of downstream cytokines such as IL-1β and IL-18. Then, cells release intracellular substances such as lactate dehydrogenase (LDH), mediating cell damage. During cell injury, ion channel opening induced by ATP release and binding purine receptor P2X7 is one of the classic pathways of NLRP1 inflammasome activation (Meng et al., 2014). More and more studies have shown the important pathophysiological function of P2X7 receptor (P2X7R) in central nervous system diseases (Divirgilio, 2007). Recent studies have found increased expression of NLRP1, ASC and Caspase-1 in STZ-induced diabetic cortical neurons (Meng et al., 2014). Therefore, P2X7R/NLRP1/caspase-1 mediated neuronal injury plays an important role in diabetic cognitive impairment and eventually inflammatory cascade reaction Bartlett et al. (2014), Sperlágh and Illes (2014), which may provide a new target for the prevention and treatment of diabetic cognitive impairment.

Naofucong is a compound preparation based on traditional Chinese medicine theory and modern pharmacology. NFC consisted of ginseng, salvia, polygonum multiflorum, leeches, poria, berberine, and calamus. Among these herbs, it has been reported that ginseng has beneficial effects in diabetes, and Panax ginseng roots extracts and Polygonum multflorum extracts could improve the learning and memory ability (Jing et al., 2020). Clinical practice has shown that it can significantly improve cognitive dysfunction in diabetics. Animal experiments have also shown that Naofucong can improve the learning and memory function and shorten the incubation period and duration of water maze in diabetic rats through up-regulating the expression of insulin-like growth factor-1 (IGF-1) and glial fibrillary acidic protein (GFAP) and decreasing the expression of nuclear factor-κB (NF-κB) in hippocampus (Zhang et al., 2004; Jing et al., 2020). Our previous studies showed that Naofucong could play a role in improving high glucose-induced neuronal injury (Jing et al., 2016). However, its specific mechanism is unknown. In this study, high glucose-induced HT22 hippocampal neurons were used to determine Naofucong-containing serum neuronal protective effects. Lentiviruses knock out of TXNIP and P2X7R was used to determine that protective effects of Naofucong was related to inflammatory response and P2X7R/NLRP1/caspase-1 mediated neuronal injury. This study can provide novel evidence to support the future clinical use of Naofucong.

## Materials and Methods

### Preparation of Naofucong

Naofucong (NFC) Granules consists of the following dried raw materials: *Panax ginseng* C.A.Mey. [Araliaceae; Ginseng radix et rhizoma], *Salvia miltiorrhiza* Bge. [Lamiaceae; Salviae miltiorrhizae radix et rhizoma], *Polygonum multiflorum* Thunb. [Polygonaceae; Polygoni multiflori radix], *Poria cocos (Schw.)* Wolf [Polyporcee; Poria], *Coptis chinensis* Franch. [Ranunculaceae; Coptidis rhizoma], *Whitemania pigra* Whitman [Hirudinidae; Hirudo] and *Acorus tatarinowii* Schott. [Araceae; Acori tatarinowii rhizoma] (1:3:3:1:1:1:1). These 7 herbs were purchased from Medicinal Materials Company of Beijing Tongrentang (Batch Number: X,157,631), processed by Beijing Kangrentang Pharmaceutical Co., LTD, China, which was prepared into an aqueous solution.

For preparation of the NFC decoction, the mixed crude drugs were soaked with stilled water at room temperature (25°C) for 2 h. For the first decoction, the drugs were refluxed with 10-fold of water (1:10, *w/v*) for 1.5 h before filtered. For the second decoction, the drug residues were refluxed with eightfold of water (1:8, *w/v*) before filtered. The two decoctions were then mixed together and concentrated in vacuum. The concentrated decoction was freeze-dried with an extraction yield of 19%. Then the NFC extract was stored under −80°C and well suspended in water before use.

### UPLC/Q-TOF-MS Analysis

The freeze-dried powders of NFC decoction water extract (20 mg) was dissolved in 10 ml of distilled water and then filtered with a 0.22 mm membrane before analysis. UPLC/MS analysis was performed on a UPLC system coupled with XEVO G2 Q-TOF mass spectrometer via an ESI source (Waters Corp. Milford, MA). For UPLC separation, 2 μL of sample solution was injected into an ACQUITY HSS T3 C_18_ column (100 × 2.1 mm, 1.7 μm, Waters). The mobile phase consisted of ACN (A) and water containing 0.1% (v/v) formic acid (B). Linear gradient elution was applied (0–5min, 5–30% A; 5–10min, 30–40% A; 10–20min, 40–65% A; 20–25min, 65–90% A) at a flow rate of 0.4 ml/min. The column temperature was 45°C. For MS detection, accurate mass was maintained by the LockSpray interface of sulfadimethoxine (309.0658 [MH]^−^). The operating parameters in negative ion mode were as follows: capillary voltage, 3.0 kV; cone voltage, 30 V; desolvation gas flow rate, 750 L/h; source temperature, 120°C; desolvation temperature, 350°C. MS data were acquired in centroid mode and processed by MassLynx software (Waters, version 4.1).

### Preparation of Animals and Naofucong-Containing Serum

SPF grade male SD rats (weighing 200–220 g, No. SCXK 2007–004) were provided by Vital River Laboratory Animal Technology Co. Ltd. (Beijing, China), and were kept in the clean animal feeding room of the animal experimental center, with a humidity of 60% and a temperature of 20–22°C. Rats were kept in a cage and fed freely. After 3 days of adaptive feeding, intragastric administration (Naofucong 4.667 g/kg) was performed, twice per day, for 3 days. After administration, 5 ml/kg 1% pentobarbital sodium was injected intraperitoneally, blood was collected from the abdominal aorta, and Naofucong-containing serum was obtained by centrifugation. Serum was filtered through 0.22 μm filters, inactivated at 56°C for 30 min. This study was performed under the supervision of the Animal Care and Use Committee of Peking Union Medical College Hospital.

### Group Processing of Cell Experiments

HT22 (immortalized mouse hippocampal neuronal) cells were kindly provided by Cell bank, Institute of Basic Medicine, Peking Union Medical College. HT22 cells were maintained in DMEM (dulbecco’s modified eagle medium)/high-glucose media (Hyclone, Logan, Utah) containing 10% fetal calf serum (Hyclone, Logan, Utah) and were incubated at 5% CO_2_/95% O_2_ incubation at 37°C. Cells were treated with control (Con, 5.5 mmol/L of glucose) or high-glucose (HG, 75 mmol/L of glucose) medium for 48 h. Besides, 10% Naofucong-containing serum (NFC) and N-acetyl-L-cysteine (NAC, 10 mmol/L) were carried out in high-glucose medium for 48 h.

### Preparation of RNAi Lentivirus Clones

The linearized vector was obtained by restriction enzyme digestion; The primers were annealed to prepare the target fragment; The primers were designed to add restriction sites at both ends of the primers, and after annealing, the primer contained the same restriction sites as the two ends of the linearized clone vector. Linearized carrier and annealing product were used to prepare the reaction system, and the products were directly transformed. Monoclones were selected from the plate for PCR identification, and the positive clones were sequenced and the results were analyzed. The high purity plasmid was obtained by expanding culture and extraction of the correct clone bacteria liquid.

### Biochemical Assay

After treatment of cells according to experimental grouping requirements, the supernatant of cells was collected for the detection of Cell Counting Kit-8 (CCK-8, C0037, Beyotime, China), Lactate dehydrogenase (LDH, C0017, Beyotime, China), Interleukin-1β (IL-1β, PI301, Beyotime, China) and Interleukin-18 (IL-18, PI553, Beyotime, China). The experimental process was carried out according to instructions.

### TUNEL Apoptosis Assay

One Step TUNEL Apoptosis Assay Kit (C1088, Beyotime, China) is a sensitive, rapid and simple method for apoptosis detection. For cells that have been fixed, the apoptotic cells presenting green fluorescence can be detected by fluorescence microscope after washing after one-step staining. The experimental process was carried out according to instructions.

### Real-Time PCR

Total RNA was extracted with Trizol kit in one step (15,596,026, Invitrogen, United States). RNA integrity of each sample was detected by formaldehyde denatured gel electrophoresis. The content of total RNA in each sample was determined by ultraviolet spectrophotometer. According to literature, PCR amplification primers were designed by existing cDNA sequences in gene bank, and PCR amplification conditions of various genes were set according to their characteristics to perform real-time PCR detection. The reaction conditions were as follows: pre-denaturation at 94°C for 5 min, 30 cycles of denaturation at 94°C for 30 s, annealing at 54.5°C for 30 s and extension at 72°C for 30 s, and a final extension at 72°C for 10 min. SDS2.2 fluorescence quantitative operation technology data analysis software was used to process and analyze the data. *β*-actin was considered as the internal reference of the genes. The expression of target genes was calculated based on the 2^−ΔΔCt^ method, with ΔCt obtained using the following formula: ΔCt = Ct (target gene) −Ct (β-actin), and ΔΔCt = ΔCt (the experiment group) −ΔCt (the control group). The experiment was conducted in triplicates. The primer sequences for RT-PCR assay were summarized in Supplementary Table S1.

### Western Blot

Cells were added into lysate for homogenization (P0013B, Beyotime biotechnology, Shanghai, China), and centrifuged for supernatant. Then, protein concentration was determined by BCA (23,250, Thermo Fisher Scientific, United States). The supernatant containing 50 μg protein was separated by 8, 10, or 12% SDS-PAGE electrophoresis and transferred to cellulose nitrate film. The non-specific binding was reduced by using TBS-T containing 5% skim milk. Incubate with different primary antibodies (Dilution concentration is 1:1,000), and then incubate with secondary antibodies (Dilution concentration is 1:4,000), and test with ECl kit (32,109, Thermo Fisher Scientific, United States). The strips were scanned and quantified using a computer image analysis system. Detailed information of antibodies was shown in Supplementary Table S2.

### Statistical Analyses

Data are presented as mean ± SEM, calculated using SPSS 17.0 software (SPSS Inc. Chicago, IL, United States). Differences were analyzed using one-way ANOVA followed by Bonferroni post hoc test or unpaired two-tailed Student’s *t*-test with SPSS 17.0 software. *p* < 0.05 was considered statistically significant.

## Results

### Chemical Profiling of NFC

UPLC/Q-TOF-MS analysis was employed to characterize the chemical composition of NFC. A total of 29 peaks (1–29) were putatively identified by comparing their high-resolution MS data. These compounds have covered most of the main peaks in the chromatogram and different kinds of constituents were involved (Table 1, Supplementary Figure S1; Supplementary Table S3).

**TABLE 1:** Related chromatographic mass spectrometric data of compound in naofucong granule.

No	Rt	*m/z*	Proposed fomula	ppm	MS^n^	Identification
1	5.23	197.0455	C_9_H_9_O_5_	1.08	MS^2^ [197]179; MS^3^ [179]135	Tanshinol
2	13.67	342.1705	C_20_H_24_NO_4_	−1.49	[342]297,298,265,311; [297]265,282	Magnoflorine
3	14.76	417.0830	C_20_H_17_O_10_	1.36	MS^2^ [417]373,175,197; MS^3^ [373]175,197	Salvianolic acid G
4	15.15	537.1037	C_27_H_21_O_12_	0.95	MS^2^ [537]339,295; MS^3^ [339]295,321,185	Lithospermic acid
5	15.17	557.1301	C_27_H_25_O_13_	1.16	MS^2^ [557]313,243,211,405,285	Piceatannol 4′-galloylglucoside
6	15.69	717.1466	C_36_H_29_O_16_	1.54	MS^2^ [717]519,321; MS^3^ [519]321,339	Salvianolic acid L
7	16.02	359.0774	C_18_H_15_O_8_	1.25	MS^2^ [359]161,179(24),197,223(13); MS^3^ [161]133	Rosmarinate
8	17.24	717.1458	C_36_H_29_O_16_	0.8	MS^2^ [717]519,321; MS^3^ [519]321,339	Salvianolic acid L
9	17.28	322.1079	C_19_H_16_NO_4_	0.52	[322]307,308,294	Thalifendine
10	18.04	493.1138	C_26_H_21_O_10_	0.91	MS^2^ [493]295; MS^3^ [295]159,185,277,157	Salvianolic acid A
11	18.17	493.1138	C_26_H_21_O_10_	0.88	MS^2^ [493]295; MS^3^ [295]159,185,277,157	Salvianolic acid A
12	19.34	338.1372	C_20_H_20_NO_4_	−1.53	[338]323,324,294; [323]294,307,308	Jatrorrhizine
13	19.57	338.1392	C_20_H_20_NO_4_	0.52	[338]323,324,294; [323]294,307	Columbamine
14	20.13	336.1215	C_20_H_18_NO_4_	−1.51	[336]321,320,308; [321]292,293	Berberine
15	20.93	320.0904	C_19_H_14_NO_4_	−1.11	[320]292,290,293; [292]277,264	Coptisine
16	22.59	352.1549	C_21_H_22_NO_4_	−2.00	[352]337,336,338; [337]308,320	Palmatine
17	23.19	336.1214	C_20_H_18_NO_4_	−1.67	[336]321; [321]292,318	Epiberberine
18	31.19	497.3273	C31H45O6	1.12	MS^2^ [497]419,435,420,417,269	Poricoic acid A
19	32.75	269.0455	C15H9O5	1.08	MS^2^ [269]225,269,226,241,201	Emodin or Aloe-emodin
20	35.34	469.3324	C30H45O4	1.13	MS^2^ [469]423,407,337,333,409	3,16-Dihydroxylanosta-7,9 (11),24-trien-21-oic acid
21	35.99	471.3481	C30H47O4	1.19	MS^2^ [471]409,337,410,425,407	16 -Hydroxytrametenolic acid
22	36.41	481.3325	C31H45O5	1.28	MS^2^ [481]412,421,403,344,382	Poricoic acid C
23	37.13	297.1476	C19H21O3	0.28	MS^2^ [297]279,251; MS3 [279]279,264	Cryptotanshinone
24	37.36	529.3534	C32H49O6	1.02	MS^2^ [529]453,469,511,451,470	3-O-acetyl-16,26-dihydroxytrametenolic acid
25	37.39	483.3482	C31H47O4	1.35	MS^2^ [483]437,337,421; MS^3^ [437]421	Dehydrotumulosic acid
26	40.16	481.3325	C31H45O4	1.25	MS^2^ [481]311,388,403,335,421	Poricoic acid C
27	41.1	529.3536	C32H49O6	1.27	MS^2^ [529]483,460,393,392,461	3-O-acetyl-16, 27-dihydroxytrametenolic acid
28	41.15	483.3482	C31H47O5	1.32	MS^2^ [483]437,421,423; MS3 [437]421	Dehydrotumulosic acid
29	46.82	527.3742	C33H51O5	1.11	MS^2^ [527]509,481,511,483	Pachymic acid

Naofucong reduced high glucose-induced HT22 hippocampal neuron cell damage.

**FIGURE 1:**
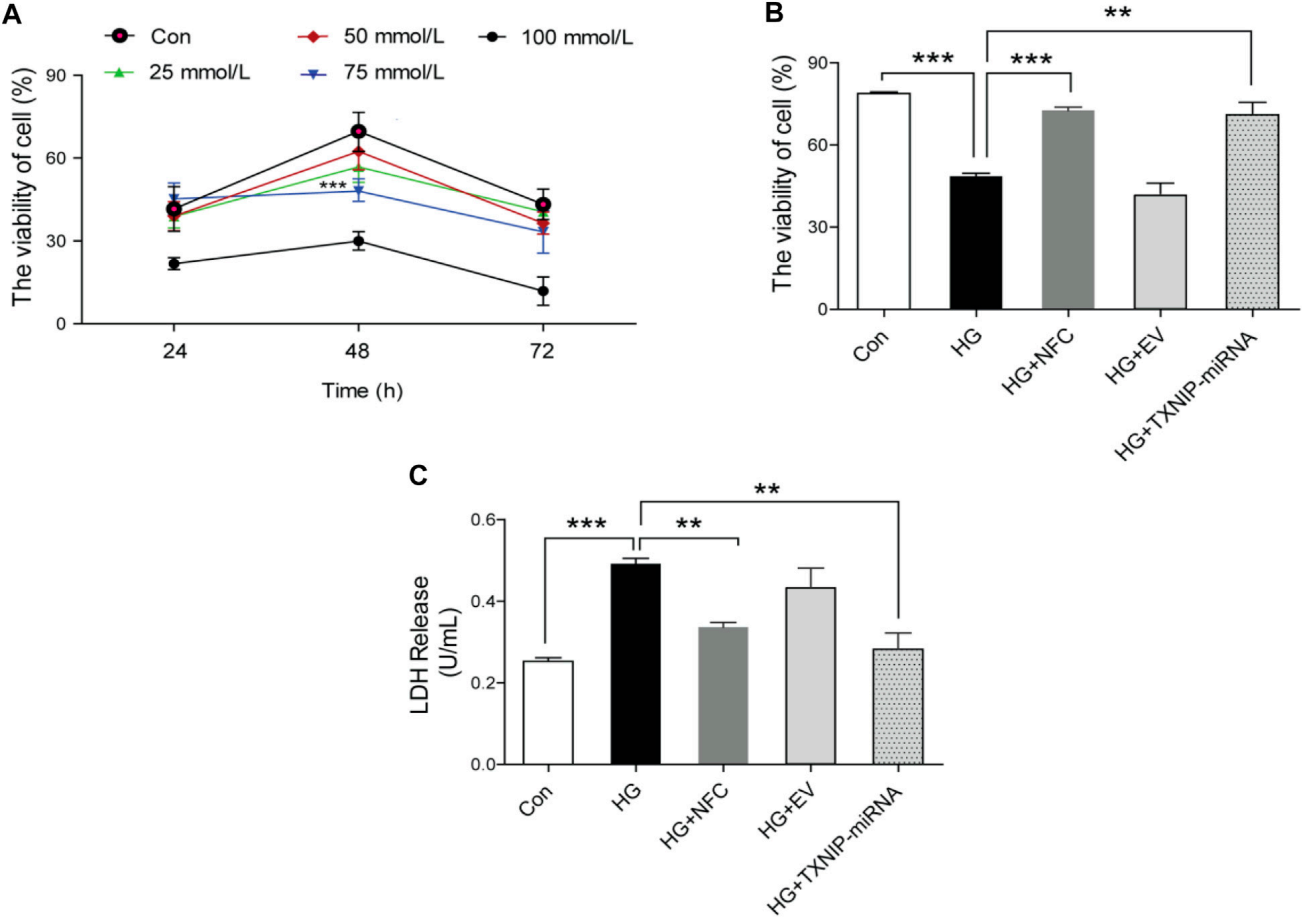
Naofucong reduced high glucose-induced HT22 hippocampal neuron cell damage. **(A)** The activity of HT22 cells cultured with high glucose at different concentrations and at different time points, ^***^ *p* < 0.001, 75 mmol/L vs. Con. **(B, C)** HT22 cell activity and LDH release level under high glucose conditions (75 mmol/L and 48 h), NFC or lentivirus knockout of TXNIP treatment. Values are means ± SEM, *n* = 5 per group, ^*^ *p* < 0.05, ^**^ *p* < 0.01, ^***^ *p* < 0.001.

In this study, HT22 hippocampal neuron cells were used to determine optimal cell model stimulation conditions after different time and different concentration (Figure 1A) of high glucose stimulation. According to results of this study and literature reports Zhang et al. (2018), 75 mmol/L and 48 h were finally determined as optimal stimulus conditions. At the same time, the study found that activity of HT22 cells decreased and LDH level increased under high glucose culture, and NFC could significantly reverse above trend. In addition, lentivirus knockout of TXNIP (a key molecule in inflammatory process), also significantly increased cell activity and decreased LDH release (Figures 1B,C). These results suggest that NFC significantly ameliorates high glucose-induced HT22 cell damage and that this effect may be related to its involvement in inflammatory process.

Naofucong decreased apoptosis, IL-1β and IL-18 levels in high glucose-induced HT22 hippocampal neuron cells.

**FIGURE 2:**
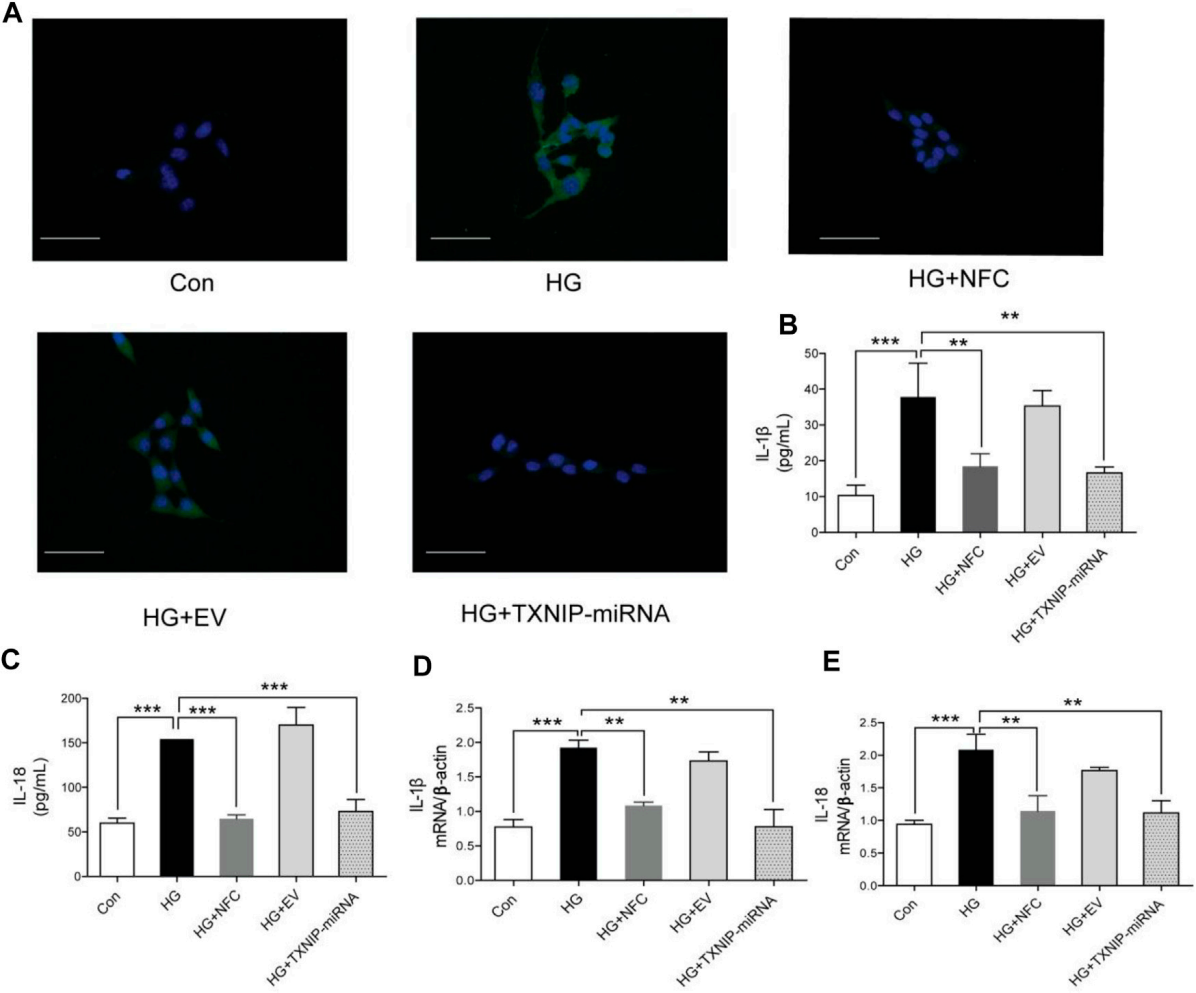
Naofucong decreased apoptosis, IL-1β and IL-18 levels in high glucose-induced HT22 hippocampal neuron cells. **(A)** Morphological photomicrographs of TUNEL staining. TUNEL positive staining cells were labeled as green, scale bar = 30 μm. **(B, C)** Effects of NFC on IL-1β and IL-18 levels of HT22 cells in high glucose. **(D, E)** Effects of NFC on IL-1β and IL-18 mRNA levels of HT22 cells in high glucose. Values are means ± SEM, *n* = 5 per group, ^*^ *p* < 0.05, ^**^ *p* < 0.01, ^***^ *p* < 0.001. Naofucong suppressed NLRP1/caspase-1 mediated neuronal injury in high glucose-induced HT22 hippocampal neuron cells.

In this study, it was found that apoptosis of HT22 cells was significantly increased in HG group, while NFC and TXNIP-miRNA could significantly reduce number of apoptotic cells (Figure 2A). Moreover, high glucose significantly increased expression levels of IL-1β and IL-18 proteins in HT22 cells, and NFC and TXNIP-miRNA significantly reversed above trends (Figures 2B,C). And, NFC and TXNIP-miRNA also significantly reversed elevated IL-1β and IL-18 mRNA levels in HT22 cells induced by high glucose (Figures 2B,C). These data showed that there was inflammatory response activation in HT22 cells induced by high glucose, and NFC could significantly inhibit inflammatory response.

**FIGURE 3:**
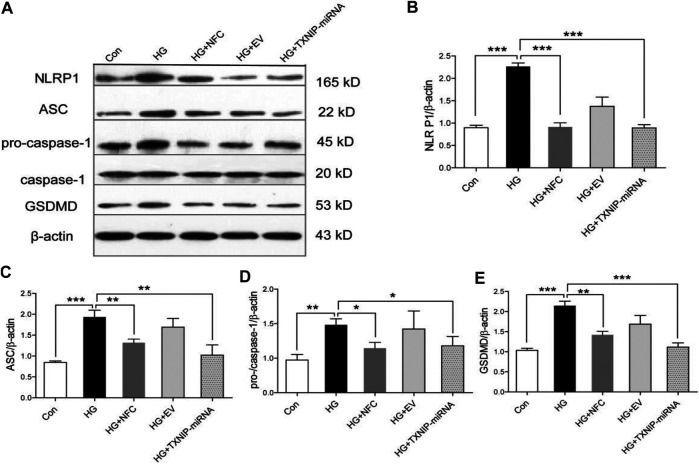
Naofucong suppressed NLRP1/caspase-1 mediated neuronal injury in high glucose-induced HT22 hippocampal neuron cells. **(A)** Effects of NFC on NLRP1, ASC, pro-caspase-1, caspase-1, and GSDMD levels of HT22 cells in high glucose. **(B–E)** Representative protein levels of NLRP1, ASC, pro-caspase-1, caspase-1, and GSDMD levels of HT22 cells in high glucose were assessed by western blotting using specific antibodies. Values are means ± SEM, *n* = 4 per group, ^*^ *p* < 0.05, ^**^ *p* < 0.01, ^***^ *p* < 0.001. Naofucong suppressed P2X7-induced neuronal injury in high glucose-induced HT22 hippocampal neuron cells.

In this study, the expression levels of key proteins in NLRP1/caspase-1 mediated neuronal injury were detected. The results showed that several key proteins in NLRP1/caspase-1 mediated neuronal injury were significantly elevated in high glucose-induced HT22 cells, including NLRP1, ASC, pro-caspase-1, caspase-1 and GSDMD (Figure 3A). And, NFC and TXNIP-miRNA significantly reduced expression levels of these proteins (Figures 3B–E). These results showed that activation of NLRP1/caspase-1 mediated neuronal injury existed in HT22 cells cultured with high glucose, the activation of NLRP1/caspase-1 mediated neuronal injury was related to TXNIP, and NFC could play a role in inhibiting NLRP1/caspase-1 mediated neuronal injury.

**FIGURE 4:**
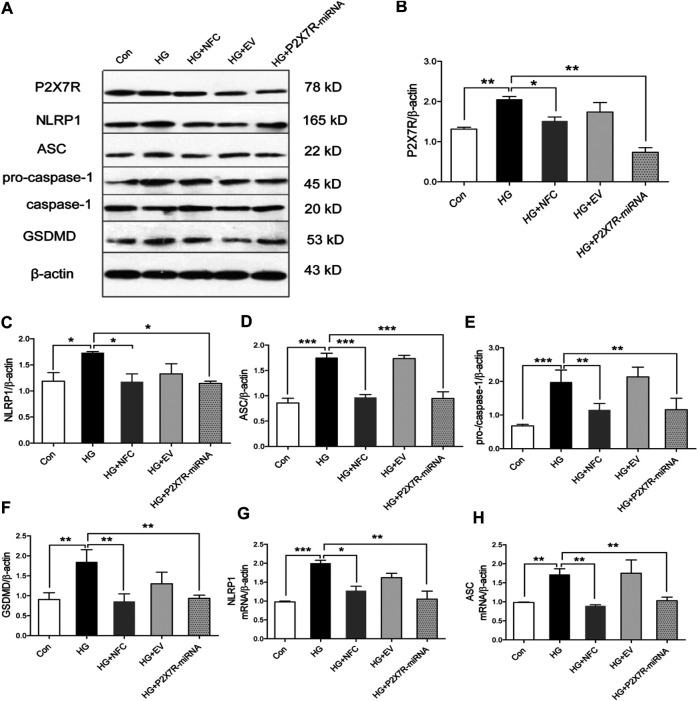
Naofucong suppressed P2X7-induced neuronal injury in high glucose-induced HT22 hippocampal neuron cells. **(A)** Effects of NFC on P2X7R, NLRP1, ASC, pro-caspase-1, caspase-1, and GSDMD levels of HT22 cells in high glucose. **(B–F)** Representative protein levels of P2X7R, NLRP1, ASC, pro-caspase-1, caspase-1, and GSDMD levels of HT22 cells in high glucose were assessed by western blotting using specific antibodies. **(G, H)** Effects of NFC and P2X7R-miRNA on NLRP1 and ASC mRNA levels of HT22 cells in high glucose. Values are means ± SEM, *n* = 4 per group, ^*^ *p* < 0.05, ^**^ *p* < 0.01, ^***^ *p* < 0.001.

In order to explore roles of P2X7 in NFC improving pyroptosis of high glucose-induced HT22 cells, P2X7R was knocked out in this study. The results showed that in HG group, P2X7R significantly increased and NFC could significantly reduce P2X7R level (Figures 4A,B). At the same time, P2X7R-miRNA can significantly reverse rising trend of key proteins in HG group and inhibit NLRP1/caspase-1 mediated neuronal injury (Figures 4C–F). And, NFC and P2X7R-miRNA also significantly reversed elevated NLRP1 and ASC mRNA levels in HT22 cells induced by high glucose (Figures 4G,H). These results indicated that P2X7 was involved in process of NFC suppressing NLRP1/caspase-1 mediated neuronal injury in high glucose-induced HT22 cells.

Oxidative stress was involved in process of NFC suppressing P2X7/NLRP1/caspase-1 mediated neuronal injury in high glucose-induced HT22 cells.

**FIGURE 5:**
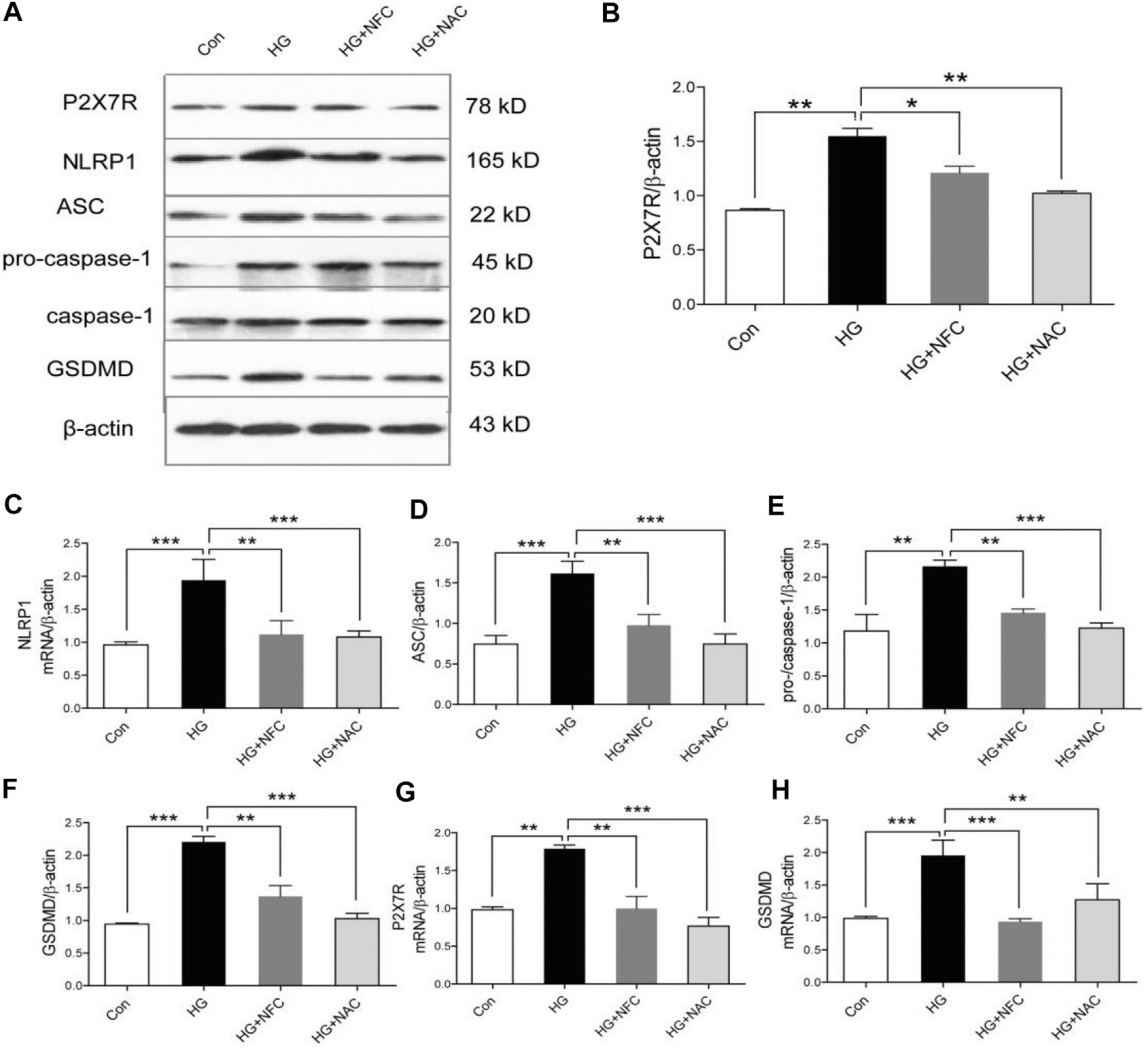
Oxidative stress was involved in process of NFC suppressing P2X7/NLRP1/caspase-1 mediated neuronal injury in high glucose-induced HT22 cells. **(A)** Effects of NAC on P2X7R, NLRP1, ASC, pro-caspase-1, caspase-1, and GSDMD levels of HT22 cells in high glucose. **(B–F)** Representative protein levels of P2X7R, NLRP1, ASC, pro-caspase-1, caspase-1, and GSDMD levels of HT22 cells in high glucose were assessed by western blotting using specific antibodies. **(G, H)** Effects of NFC and NAC on NLRP1, and ASC mRNA levels of HT22 cells in high glucose. Values are means ± SEM, *n* = 4 per group, ^*^ *p* < 0.05, ^**^ *p* < 0.01, ^***^ *p* < 0.001.

In order to explore roles of oxidative stress in high glucose-induced neuronal injury of HT22 cells, NAC (an oxidative stress inhibitor) was added in this study as a control drug with NFC. NAC can significantly reverse rising trend of P2X7R and key proteins in HG group and inhibit NLRP1/caspase-1 mediated neuronal injury (Figures 5A–F). And, NFC and NAC also significantly reversed elevated P2X7R and GSDMD mRNA levels in HT22 cells induced by high glucose (Figures 5G,H). These results indicated that HT22 cells cultured in high glucose had an internal environment with elevated oxidative stress, which could promote P2X7/NLRP1/caspase-1 mediated neuronal injury, while NFC and NAC could reduce oxidative stress and thus alleviate neuronal injury.

## Discussion

In this study, HT22 hippocampal neurons were used for high-glucose stimulation to construct a cell damage model, and Naofucong-containing serum was used to determine neuronal protective effect of Naofucong. Then, lentiviruses were used to knock out TXNIP and P2X7R, respectively, so as to determine that protective effects of Naofucong was related to inflammatory response and P2X7/NLRP1/caspase-1 mediated neuronal injury. Finally, NAC was also used to inhibit oxidative stress, so as to determine that oxidative stress is an important starting factor for P2X7/NLRP1/caspase-1 mediated neuronal injury of HT22 cells cultured with high glucose.

Naofucong is a compound preparation based on traditional Chinese medicine theory and modern pharmacology. It is rich in a variety of intelligence and neuroprotective ingredients. It has effects of tonifying kidney and invigorating spleen, nourishing blood and promoting blood circulation, and improving cognitive function. It has a good effect on patients with mild cognitive dysfunction of spleen and kidney function deficiency, phlegm and blood stasis (Zhang et al., 2004; Jing et al., 2020).

The dosage in this study was determined based on our clinical dosage and previous animal experiments (Jing et al., 2016; Jing et al., 2020). The dose-effect of drug-containing serum is also determined by dose and serum concentration of donor animal. Since serum concentration in this cell culture system has been limited to fixed condition of 10%, blood drug concentration is mainly adjusted according to dose given by donor animal. The dose of Chinese herb *in vitro* test is determined according to dose-effect relationship *in vivo* test under condition that effective components of Chinese herb are not completely clear (Heinrich et al., 2020). The dose used in this study is equivalent to the dose used in human clinical practice.

Hippocampal neurons are main cells in brain for learning and memory, and HT22 cells have been used as well established *in vitro* cellular models for neurodegenerative disorders such as AD. This cell lines has functional cholinergic properties related to the cognitive defects of AD (Liu et al., 2009). In this study, HT22 hippocampal neuron cells were used to determine optimal cell model stimulation conditions after different time and different concentration of high glucose stimulation. According to results of this study and literature reports, 75 mmol/L and 48 h were finally determined as optimal stimulus conditions. Thioredoxin-interacting protein (TXNIP) was a type of thioredoxin binding protein (TRX), which mediated oxidative stress, inhibited cell proliferation, and induced apoptosis by inhibiting function of thioredoxin system. TXNIP is also a central molecule in inflammatory process Singh et al. (2017), and in this study, lentivirus knockout of TXNIP was used to determine relationships between protective effects of Naofucong and inflammatory response. These results suggest that NFC significantly ameliorates high glucose-induced HT22 cell damage. And there was inflammatory response activation in HT22 cells induced by high glucose, and NFC could significantly inhibit inflammatory response.

P2X7/NLRP1/caspase-1 pathway is an important pro-inflammatory pathway, which depends on caspase-1 activation and followed by inflammatory cascade (Tan et al., 2014). In this study, the expression levels of key proteins in NLRP1/caspase-1 mediated neuronal injury were detected. These results showed that activation of NLRP1/caspase-1 mediated neuronal injury existed in HT22 cells cultured with high glucose, the activation of NLRP1/caspase-1 mediated neuronal injury was related to TXNIP, and NFC could play a role in inhibiting NLRP1/caspase-1 mediated neuronal injury. During cell injury, ion channel opening induced by ATP release and binding purine receptor P2X7 is one of the classic pathways of NLRP1 inflammasome activation. P2X7R, a member of the P2X family of purine receptors, is a type of ion channel that is permeable to potassium, sodium and calcium (Kasuya et al., 2016). It was found that P2X7R binds to NLRP1 inflammasome through Pannexin 1 (Pannexin 1) in the cytoplasm of neurons, inducing caspase-1 activation and IL-1β maturation and release (Meng et al., 2014). In order to explore roles of P2X7 in NFC improving pyroptosis of high glucose-induced HT22 cells, P2X7R was knocked out in this study. These results indicated that P2X7 was involved in process of NFC suppressing NLRP1/caspase-1 mediated neuronal injury in high glucose-induced HT22 cells.

There are many mechanisms of activation of inflammasome complexes in central nervous system. Hyperglycemia causes excessive production of superoxide anions in mitochondria, which will lead to oxidative stress in tissues and cells and eventually lead to various complications of diabetes (Maiese et al., 2007). Many studies have shown that, ROS may be involved in the activation of NLRP1, thereby enhancing the inflammatory response (Xu et al., 2013). Recent studies have shown that hyperglycemia increases the production of ROS in myocardial cells, which in turn upregulates NF-κB and TXNIP. NF-κB in turn upregulates IL-1β precursor, and IL-18 precursor. TXNIP activates Caspase-1 by changing the structure of NLRP1 (Fann et al., 2013), (Masters et al., 2012). The activated Caspase-1, on the one hand, cleases Gasdermin D to form a peptide containing the active domain of nitrogen end of Gasdermin D, which induces the perforation and rupture of myocardial cell membrane, eleases contents, and causes inflammatory reaction. On the other hand, activated caspase-1 excises the precursors of IL-1β and IL-18 to form active IL-1β and IL-18, which are released outside the cell to recruit inflammatory cell aggregation and amplify the inflammatory response (Kıçık et al., 2020). In order to explore roles of oxidative stress in high glucose-induced neuronal injury of HT22 cells, NAC (an oxidative stress inhibitor) was added in this study as a control drug with NFC. These results indicated that HT22 cells cultured in high glucose had an internal environment with elevated oxidative stress, which could promote pyroptosis, while NFC and NAC could reduce oxidative stress and thus alleviate pyroptosis.

The existing problems and future study directions were also summarized as follows. Firstly, because of complicated composition of NFC, only parts of major compounds were identified presently. The key effective constituents remain unknown. Secondly, the complexity of components of Chinese herb determines that effect of Chinese herb on body is a comprehensive embodiment of therapeutic effect of multiple components, multiple targets and multiple channels, while the dose-effect relationship of Chinese herb is still in the stage of accumulation of experience and faces many bottlenecks. At the same time, some influencing factors of Chinese herb itself, such as place of origin, time of collection, processing methods, etc., also need to be considered. Moreover, the drug-containing serum itself does have certain limitations, such as irregular absorption, low bioavailability, and often not obvious dose-effect relationship. The biggest limitation of this experiment is that there is no design of high, medium and low dose drug gradient, and only a single dose was used, and there was a lack of comparison between doses of different concentrations. And, more drug concentration gradients need to be designed in the future to further explore the drug dose-effect relationship. The most appropriated dose of NFC decoction for clinical use still needs more consideration (such as long-term safety) and requires further investigations. Despite this, we should delve deeper to perform further mechanism studies for better understanding the therapeutic effects of NFC decoction and applying it into the management of diabetic cognitive dysfunction.

## Conclusion

Naofucong significantly improves high glucose-induced HT22 hippocampal neuron injury, which is related to suppress P2X7/NLRP1/caspase-1 pathway. This provides novel evidence to support the future clinical use of Naofucong. However, there is a major defect in current study, which is that the dose is not clearly defined. In this study, there is a lack of effect comparison of multiple doses, which also affects clinical application of NFC. In our follow-up study, we will conduct multiple dose studies to find optimal dose of NFC.

## Data Availability

The original contributions presented in the study are included in the article/Supplementary Material, further inquiries can be directed to the corresponding author.

## References

[B1] Bartlett R. Stokes L. Sluyter R. (2014). The P2X7 Receptor Channel: Recent Developments and the Use of P2X7 Antagonists in Models of Disease. Pharmacol. Rev. 66, 638–675. 10.1124/pr.113.008003 24928329

[B2] Divirgilio F. (2007). Liaisons Dangereuses: P2X7 and the Inflammasome. Trends Pharmacological Sciences 28, 465–472. 10.1016/j.tips.2007.07.002 17692395

[B3] Doitsh G. Galloway N. L. K. Geng X. Yang Z. Monroe K. M. Zepeda O. (2014). Cell Death by Pyroptosis Drives CD4 T-Cell Depletion in HIV-1 Infection. Nature 505, 509–514. 10.1038/nature12940 24356306 PMC4047036

[B4] Fann D. Y. Lee S. Y. Manzanero S. Tang S. C. Gelderblom M. Chunduri P. (2013). Intravenous Immunoglobulin Suppresses NLRP1 and NLRP3 Inflammasome-Mediated Neuronal Death in Ischemic Stroke. Cell Death Dis. 4, e790. 10.1038/cddis.2013.326 24008734 PMC3789184

[B5] Heinrich M. Appendino G. Efferth T. Fürst R. Izzo A. A. Kayser O. (2020). Best Practice in Research - Overcoming Common Challenges in Phytopharmacological Research. J. ethnopharmacology 246, 112230. 10.1016/j.jep.2019.112230 31526860

[B7] Jing G.-C. Liu D. Liu Y.-Q. Zhang M.-R. (2020). Nao-Fu-Cong Ameliorates Diabetic Cognitive Dysfunction by Inhibition of JNK/CHOP/Bcl2-mediated Apoptosis In Vivo and In Vitro. Chin. J. Nat. medicines 18, 704–713. 10.1016/s1875-5364(20)60009-7 32928514

[B6] Jing G.-c. Zhang M.-r. Ji C. Zuo P.-p. Liu Y.-q. Gu B. (2016). Effect of Chinese Herbal Compound Naofucong (脑复聪) on the Inflammatory Process Induced by High Glucose in BV-2 Cells. Chin. J. Integr. Med. 22, 832–839. 10.1007/s11655-016-2256-0 27225293

[B8] Kasuya G. Fujiwara Y. Takemoto M. Dohmae N. Nakada-Nakura Y. Ishitani R. (2016). Structural Insights into Divalent Cation Modulations of ATP-Gated P2X Receptor Channels. Cel Rep. 14, 932–944. 10.1016/j.celrep.2015.12.087 26804916

[B9] Kıçık A. Tüzün E. Erdoğdu E. Bılgıç B. Tüfekçıoğlu Z. Öztürk-Işik E. (2020). Neuroinflammation Mediators Are Reduced in Sera of Parkinson's Disease Patients with Mild Cognitive Impairment. Noro psikiyatri arsivi 57, 15–17. 32110144 10.29399/npa.23624 PMC7024832

[B10] Liu J. Li L. Suo W. Z. (2009). HT22 Hippocampal Neuronal Cell Line Possesses Functional Cholinergic Properties. Life Sci. 84, 267–271. 10.1016/j.lfs.2008.12.008 19135458

[B11] Maiese K. Chong Z. Z. Shang Y. C. (2007). Mechanistic Insights into Diabetes Mellitus and Oxidative Stress. Curr. Med. Chem. 14, 1729–1738. 10.2174/092986707781058968 17627510 PMC2447161

[B12] Masters S. L. Gerlic M. Metcalf D. Preston S. Pellegrini M. O’Donnell J. A. (2012). NLRP1 Inflammasome Activation Induces Pyroptosis of Hematopoietic Progenitor Cells. Immunity 37, 1009–1023. 10.1016/j.immuni.2012.08.027 23219391 PMC4275304

[B13] Meng X.-F. Wang X.-L. Tian X.-J. Yang Z.-H. Chu G.-P. Zhang J. (2014). Nod-like Receptor Protein 1 Inflammasome Mediates Neuron Injury under High Glucose. Mol. Neurobiol. 49, 673–684. 10.1007/s12035-013-8551-2 24014157

[B14] Palta P. Schneider A. L. C. Biessels G. J. Touradji P. Hill-Briggs F. (2014). Magnitude of Cognitive Dysfunction in Adults with Type 2 Diabetes: a Meta-Analysis of Six Cognitive Domains and the Most Frequently Reported Neuropsychological Tests within Domains. J. Int. Neuropsychol. Soc. 20, 278–291. 10.1017/s1355617713001483 24555960 PMC4132660

[B15] Park M.-K. Lee J.-W. Lee J.-C. Hwang S.-J. Roh H. W. Hong C. H. (2018). NLRP1 and NTN1, Deregulated Blood Differentially Methylated Regions in Mild Cognitive Impairment Patients. J. Mol. Neurosci. 66, 561–571. 10.1007/s12031-018-1180-5 30397880

[B16] Romeo G. R. Lee J. Shoelson S. E. (2012). Metabolic Syndrome, Insulin Resistance, and Roles of Inflammation - Mechanisms and Therapeutic Targets. Arterioscler Thromb. Vasc. Biol. 32, 1771–1776. 10.1161/atvbaha.111.241869 22815343 PMC4784686

[B17] Singh L. P. Devi T. S. Yumnamcha T. (2017). The Role of Txnip in Mitophagy Dysregulation and Inflammasome Activation in Diabetic Retinopathy: A New Perspective. JOJ Ophthalmol. 4. 10.19080/jojo.2017.04.555643 PMC5786434 29376145

[B18] Sperlágh B. Illes P. (2014). P2X7 Receptor: an Emerging Target in Central Nervous System Diseases. Trends Pharmacological Sciences 35, 537–547. 10.1016/j.tips.2014.08.002 25223574

[B20] Tan C.-C. Zhang J.-G. Tan M.-S. Chen H. Meng D.-W. Jiang T. (2015). NLRP1 Inflammasome Is Activated in Patients with Medial Temporal Lobe Epilepsy and Contributes to Neuronal Pyroptosis in Amygdala Kindling-Induced Rat Model. J. neuroinflammation 12, 18. 10.1186/s12974-014-0233-0 25626361 PMC4314732

[B19] Tan M. S. Tan L. Jiang T. Zhu X. C. Wang H. F. Jia C. D. (2014). Amyloid-β Induces NLRP1-dependent Neuronal Pyroptosis in Models of Alzheimer's Disease. Cel Death Dis. 5, e1382. 10.1038/cddis.2014.348 PMC4454321 25144717

[B21] White C. S. Lawrence C. B. Brough D. Rivers-Auty J. (2017). Inflammasomes as Therapeutic Targets for Alzheimer's Disease. Brain Pathol. 27, 223–234. 10.1111/bpa.12478 28009077 PMC8029266

[B22] Xu Y. Wang L. He J. Bi Y. Li M. Wang T. (2013). Prevalence and Control of Diabetes in Chinese Adults. Jama 310, 948–959. 10.1001/jama.2013.168118 24002281

[B24] Zhang M.-Y. Li Y. Yin S.-Y. Kong L. Liu X.-L. Yin X.-X. (2018). Sarsasapogenin Suppresses Aβ Overproduction Induced by High Glucose in HT-22 Cells. Naunyn-schmiedeberg's Arch. Pharmacol. 391, 159–168. 10.1007/s00210-017-1445-5 29275517

[B23] Zhang M. R. Guo S. S. Xu H. Y. (2004). [Study on the Mechanism of Naofucong Granule in Improving Memory of Cerebral Ischemic Mice]. Zhongguo Zhong Xi Yi Jie He Za Zhi 24, 147–149. 15015451

