# Supplementary material for: Naofucong Ameliorates High Glucose Induced Hippocampal Neuron Injury Through Suppressing P2X7/NLRP1/Caspase-1 Pathway
Source: Front Pharmacol. 2021 May 20;12:647116. doi: 10.3389/fphar.2021.647116 (PMC8173084; doi:10.3389/fphar.2021.647116)
Supplement: Supplementary file 1 [file DataSheet1.docx]

**Supplementary materials**

**Table S1 the primer sequences for RT-PCR assay**

| **Gene** | **Primer sequence (5’ to 3’)** |
| --- | --- |
| ***β-actin*** | *F：GAGATTACTGCTCTGGCTCCTA* |
|  | *R：GGACTCATCGTACTCCTGCTTG* |
| ***P2X7R*** | *F：GGCATAGCAGAGGTGACGGAGA* |
|  | *R：GCACTTGGCCTTCTGACTTGACAT* |
| ***TXNIP*** | *F：TGGACGATGTGGACGACTCTCA* |
|  | *R：GTTGTTGTTAAGGACGCACGGATC* |
| ***ASC*** | *F：ACCAGCACAGGCAAGCACTCA* |
|  | *R：GCTCCAGGTCCATCACCAAGTAGG* |
| ***GSDMD*** | *F：ATCACTGAGGTCCACAGCCAAGAG* |
|  | *R：CCACTCGGAATGCCAGGATGCT* |
| ***caspase-1*** | *F：CTGGTCTTGTGACTTGGAGGACAT* |
|  | *R：ATTGGCACGATTCTCAGCATAGGT* |
| ***IL-1β*** | *F：AAAGCCTCGTGCTGTCGG* |
|  | *R：TAAGGAGTCCCCTGGAGATTG* |
| ***NLRP1*** | *F：CAGAGGCTCAGTGGTTAGCAGTTG* |
|  | *R：AAGTGGGAAGGAGGCTGTTGGAT* |
| ***IL-18*** | *F：TGGAGACCTGGAATCAGACAACTT* |

**Table S2 Detailed information of antibodies for Western Blot**

| **Description** | **Brand** | **Product Code** | **Production Place** |
| --- | --- | --- | --- |
| P2X7 | Santa Cruz | SC-134224 | USA |
| VDUP1 | Santa Cruz | sc-166234 | USA |
| NALP1 | Santa Cruz | SC-166368 | USA |
| ASC | CST | #67824 | USA |
| GSDMDC1 | Santa Cruz | SC-393656 | USA |
| Caspase-1 | Santa Cruz | SC-392736 | USA |
| pro-caspase-1 | Abcam | ab179515 | UK |
| Anti-beta Actin | Abcam | ab8227 | UK |
| Goat Anti-Rabbit IgG | Abcam | ab6721 | UK |
| Goat Anti-Mouse IgG | Abcam | ab6788 | UK |
| Rabbit Anti-Rat IgG | Abcam | ab6734 | UK |

**Fig. S1 TIC diagram of methanol extract of naofucong (NFC) granule** (Blue is the compound that can predict structure, red is the compound that cannot predict structure temporarily)

**Table S3 Related chromatographic mass spectrometric data of compound in naofucong (NFC) granule**

| No. | Rt. | *m/z* | proposed fomula | ppm | MS^n^ |
| --- | --- | --- | --- | --- | --- |
| H1 | 2.09 | 341.1089 |  |  | MS^2^ [341]178(100),161(24),113(18); MS^3^ [178] 89(100),143(98) |
| H2 | 2.14 | 527.1609 |  |  | [527]365(100),347(29),467(20);[365]305(100),347(20) |
| H3 | 2.2 | 470.1515 |  |  | MS^2^ [480]341(100),161(24),179(13); MS^3^ [341]179(100),160(23) |
| H4 | 4.94 | 166.0861 |  |  | MS2[166]120(100),149(5),148(1);MS3[120]93(100),103(25) |
| H5 | 5.23 | 197.0455 | C_9_H_9_O_5_ | 1.08 | MS^2^ [197]179(100); MS^3^ [179]135(100),107(4),109(1) |
| H6 | 10.64 | 367.1037 |  |  | MS^2^ [367]191(100),173(37),193(15); MS^3^ [191]93(100),85(45) |
| H7 | 12.33 | 452.0810 |  |  | MS^2^ [452]367(100),173(22),389(12); MS^3^ [367]173(100),191(32) |
| H8 | 12.42 | 367.1035 |  |  | MS^2^ [367]173(100),191(94); MS^3^ [173]93(100),110(77),109(15) |
| H9 | 13.67 | 342.1705 | C_20_H_24_NO_4_ | -1.49 | [342]297(100),298(28),265(23),311(20);[297]265(100),282(13) |
| H10 | 14.09 | 405.1192 |  |  | MS^2^ [405]243(100); MS^3^ [243]149(100),225(96),215(83),137(76) |
| H11 | 14.11 | 451.1247 |  |  | MS^2^ [451]405(100),315(5); MS^3^ [405]243(100) |
| H12 | 14.76 | 417.0830 | C_20_H_17_O_10_ | 1.36 | MS^2^ [417]373(100),175(99),197(27); MS^3^ [373]175(100),197(31) |
| H13 | 15.15 | 537.1037 | C_27_H_21_O_12_ | 0.95 | MS^2^ [537]339(100),295(35); MS^3^ [339]295(100),321(45),185(13) |
| H14 | 15.17 | 557.1301 | C_27_H_25_O_13_ | 1.16 | MS^2^ [557]313(100),243(66),211(27),405(20),285(15); |
| H15 | 15.69 | 717.1466 | C_36_H_29_O_16_ | 1.54 | MS^2^ [717]519(100),321(15); MS^3^ [519]321(100),339(36) |
| H16 | 15.74 | 853.5763 |  |  | MS^2^ [853]809(100),807(87),818(44); |
| H17 | 15.9 | 500.1566 |  |  | MS^2^ [500]338(100); MS^3^ [338]323(100) |
| H18 | 16.02 | 359.0774 | C_18_H_15_O_8_ | 1.25 | MS^2^ [359]161(100),179(24),197(18),223(13); MS^3^ [161]133(100) |
| H19 | 16.53 | 739.1278 |  |  | MS^2^ [739]559(100),515(91),695(54); MS^3^ [559]515(100),335(30) |
| H20 | 16.98 | 845.4895 |  |  | MS^2^ [845]799(100),637(31); |
| H21 | 17.05 | 739.1279 |  |  | MS^2^ [739]558(100),515(63),695(40),540(25); |
| H22 | 17.24 | 717.1458 | C_36_H_29_O_16_ | 0.8 | MS^2^ [717]519(100),321(14); MS^3^ [519]321(100),339(21) |
| H23 | 17.28 | 322.1079 | C_19_H_16_NO_4_ | 0.52 | [322]307(100),308(16),294(10),279(5),295(2) |
| H24 | 18.04 | 493.1138 | C_26_H_21_O_10_ | 0.91 | MS^2^ [493]295(100); MS^3^ [295]159(100),185(29),277(22),157(18) |
| H25 | 18.17 | 493.1138 | C_26_H_21_O_10_ | 0.88 | MS^2^ [493]295(100); MS^3^[295]159(100),185(29),277(22),157(18) |
| H26 | 18.4 | 583.0834 |  |  | MS^2^ [583]537(100),515(32); MS^3^[537]356(100),291(96),493(61) |
| H27 | 19.34 | 338.1372 | C_20_H_20_NO_4_ | -1.53 | [338]323(100),324(14),294(13);[323]294(100),307(25),308(10) |
| H28 | 19.57 | 338.1392 | C_20_H_20_NO_4_ | 0.52 | [338]323(100),324(14),294(13);[323]294(100),307(25) |
| H29 | 19.88 | 766.2145 |  |  | MS^2^ [766]431(100); MS^3^ [431]269(100) |
| H30 | 20.13 | 336.1215 | C_20_H_18_NO_4_ | -1.51 | [336]321(100),320(93),308(23);[321]292(100),293(23) |
| H31 | 20.93 | 320.0904 | C_19_H_14_NO_4_ | -1.11 | [320]292(100),290(14),293(11);[292]277(100),264(32) |
| H32 | 21.47 | 845.4906 |  |  | MS^2^ [845]799(100),418(37); |
| H33 | 21.63 | 801.4922 |  |  | [801]647(100),788(91),716(83),624(80),715(72) |
| H34 | 22.03 | 599.8015 |  |  | MS^2^ [599]576(100),553(47),462(12),531(8),578(5); |
| H35 | 22.13 | 785.4998 |  |  | [785]700(100),571(82),321(78),447(45),605(45) |
| H36 | 22.24 | 459.1451 |  |  | MS^2^ [459]390(100),399(71),297(53); |
| H37 | 22.59 | 352.1549 | C_21_H_22_NO_4_ | -2.00 | [352]337(100),336(18),338(15);[337]308(100),320(22) |
| H38 | 22.93 | 829.4953 |  |  | MS^2^ [829]783(100); MS^3^ [783]475(100),637(53),619(20) |
| H39 | 23.19 | 336.1214 | C_20_H_18_NO_4_ | -1.67 | [336]321(100);[321]292(100),318(14),304(5),275(2),320(1) |
| H40 | 23.48 | 683.4377 |  |  | MS^2^[515]:471(100),455(31) |

| No. | Rt. | *m/z* | proposed fomula | ppm | MS^n^ |
| --- | --- | --- | --- | --- | --- |
| H41 | 29.07 | 811.4853 |  |  | MS^2^[755]:695(100),635(18) |
| H42 | 31.12 | 829.4951 |  |  | MS^2^[755]:695(69),633(100);MS^3^[633]:577(100),511(77) |
| H43 | 31.19 | 497.3273 | C_31_H_45_O_6_ | 1.12 | MS^2^ [497]419(100),435(38),420(32),417(26),269(11); |
| H44 | 31.66 | 785.4988 |  |  | [785]767(100),726(57),648(42),739(29),700(27) |
| H45 | 31.66 | 829.4954 |  |  | MS^2^ [829]783(100); MS^3^ [783]621(100),459(12) |
| H46 | 32.75 | 269.0455 | C_15_H_9_O_5_ | 1.08 | MS^2^ [269]225(100),269(50),226(21),241(14),201(13); |
| H47 | 34.45 | 443.2765 |  |  | MS^2^ [443]425(100),381(60),338(16); MS^3^ [425]381(100) |
| H48 | 35.34 | 469.3324 | C_30_H_45_O_4_ | 1.13 | MS^2^ [469]423(100),407(68),337(64),333(64),409(37); |
| H49 | 35.99 | 471.3481 | C_30_H_47_O_4_ | 1.19 | MS^2^ [471]409(100),337(79),410(67),425(48),407(47); |
| H50 | 36.41 | 481.3325 | C_31_H_45_O_5_ | 1.28 | MS^2^ [481]412(100),421(89),403(89),344(86),382(61); |
| H51 | 36.99 | 811.4851 |  |  | MS^2^ [811]765(100),742(68),727(23),537(21),553(21); |
| H52 | 37.13 | 297.1476 | C19H21O3 | 0.28 | MS2[297]279(100),251(80);MS3[279]279(100),264(18) |
| H53 | 37.36 | 529.3534 | C_32_H_49_O_6_ | 1.02 | MS^2^ [529]453(100),469(80),511(53),451(41),470(21); |
| H54 | 37.39 | 483.3482 | C_31_H_47_O_4_ | 1.35 | MS^2^ [483]437(100),337(81),421(69); MS^3^[437]421(100) |
| H55 | 38.05 | 485.3639 |  |  | MS^2^ [485]337(100),437(58),386(55),421(49),422(48); |
| H56 | 39.05 | 485.3274 |  |  | MS^2^ [485]441(100),467(39),353(24),442(20),440(18); |
| H57 | 40.16 | 481.3325 | C_31_H_45_O_4_ | 1.25 | MS^2^ [481]311(100),388(63),403(40),335(34),421(34); |
| H58 | 40.68 | 483.3483 |  |  | MS^2^ [483]421(100),337(43),437(24),422(17),423(15); |
| H59 | 41.1 | 529.3536 | C_32_H_49_O_6_ | 1.27 | MS^2^ [529]483(100),460(88),393(49),392(47),461(34); |
| H60 | 41.15 | 483.3482 | C_31_H_47_O_5_ | 1.32 | MS^2^ [483]437(100),421(92),423(73); MS^3^[437]421(100) |
| H61 | 45.82 | 525.3586 |  |  | MS^2^ [525]465(100); |
| H62 | 45.82 | 593.3460 |  |  | MS^2^ [593]525(100); |
| H63 | 45.82 | 525.3587 |  |  | MS^2^ [525]465(100),355(41),479(40),481(29); MS^3^[465]421(100) |
| H64 | 45.85 | 571.3644 |  |  | MS^2^ [571]486(100); |
| H65 | 46.82 | 527.3742 | C_33_H_51_O_5_ | 1.11 | MS^2^ [527]509(100),481(87),511(47),483(37); |
| H66 | 46.84 | 595.3614 |  |  | MS^2^ [595]527(100),531(2); |
